# The tumor suppressor gene RBM5 inhibits lung adenocarcinoma cell growth and induces apoptosis

**DOI:** 10.1186/1477-7819-10-160

**Published:** 2012-08-06

**Authors:** Chen Shao, Lijing Zhao, Ke Wang, Wei Xu, Jie Zhang, Baoxue Yang

**Affiliations:** 1 Department of Pathophysiology, Norman Bethune College of Medicine of Jilin University, Changchun, Jilin, 130021, China; 2 Department of Respiratory Medicine, the Second Affiliated Hospital of Jilin University, Changchun, Jilin, 130041, China; 3 Department of Digestive Medicine, China–Japan Union Hospital of Jilin University, Changchun, Jilin, 130033, China; 4 Department of Pharmacology, School of Basic Medical Sciences of Peking University, Beijing, 100191, China

**Keywords:** RBM5, Lung adenocarcinoma, Apoptosis, A549, Xenograft mice model

## Abstract

**Background:**

The loss of tumor suppressor gene (TSG) function is a critical step in the pathogenesis of human lung cancer. RBM5 (RNA-binding motif protein 5, also named H37/LUCA-15) gene from chromosome 3p21.3 demonstrated tumor suppressor activity. However, the role of RBM5 played in the occurrence and development of lung cancer is still not well understood.

**Method:**

Paired non-tumor and tumor tissues were obtained from 30 adenocarcinomas. The expression of RBM5 mRNA and protein was examined by RT-PCR and Western blot. A549 cell line was used to determine the apoptotic function of RBM5 *in vitro*. A549 cells were transiently transfected with pcDNA3.1-RBM5. AnnexinV analysis was performed by flow cytometry. Expression of Bcl-2, cleaved caspase-3, caspase-9 and PAPP proteins in A549 lung cancer cells and the A549 xenograft BALB/c nude mice model was determined by Western blot. Tumor suppressor activity of RBM5 was also examined in the A549 xenograft model treated with pcDNA3.1-RBM5 plasmid carried by attenuated *Salmonella typhi* Ty21a.

**Result:**

The expression of RBM5 mRNA and protein was decreased significantly in adenocarcinoma tissues compared to that in the non-tumor tissues. In addition, as compared to the vector control, a significant growth inhibition of A549 lung cancer cells was observed when transfected with pcDNA3.1-RBM5 as determined by cell proliferation assay. We also found that overexpression of RBM5 induced both early and late apoptosis in A549 cells using AnnexinV/PI staining as determined by flow cytometry. Furthermore, the expression of Bcl-2 protein was decreased, whereas the expression of cleaved caspase-3, caspase-9 and PARP proteins was significantly increased in the RBM5 transfected cells; similarly, expression of decreased Bcl-2 and increased cleaved caspase-3 proteins was also examined in the A549 xenograft model. More importantly, we showed that accumulative and stable overexpression of RBM5 in the A549 xenograft BALB/c nude mice model significantly inhibited the tumor growth rate *in vivo* as compared to that in the control.

**Conclusion:**

Our study demonstrates that RBM5 can inhibit the growth of lung cancer cells and induce apoptosis both *in vitro* and *in vivo*, which suggests that RBM5 might be used as a potential biomarker or target for lung cancer diagnosis and chemotherapy. Moreover, we propose a novel animal model set up in BALB/c nude mice treated with attenuated Salmonella as a vector carrying plasmids to determine RBM5 function *in vivo*.

## Background

Lung cancer is the leading cause of cancer death worldwide, with over a million deaths annually [1,2]. Lung cancer is classified into two major clinic-pathological groups, small cell lung carcinoma (SCLC) and non-small cell lung carcinoma (NSCLC). Squamous cell carcinoma, adenocarcinoma and large cell carcinoma are the major histologic types of NSCLC [3]. The proportion of lung adenocarcinoma in lung cancer has risen rapidly, which becomes the major pathological type with morbidity of 30%-40% of all lung cancer types [4]. Although tobacco smoking was considered as one of the key factors in promoting this devastating malignancy [5], the acquired genetic or epigenetic changes also played an important role in the occurrence and development of lung cancer.

Deletion within the lung cancer tumor suppressor region at chromosome 3p21.3 constitutes the earliest premalignant chromosomal aberration in human lung cancers [6]. The region of most frequent chromosomal deletion found at the earliest stage in lung cancer development houses 19 genes [7], many of which may act together as a ‘tumor suppressor group,’ representing one of the most promising opportunities for development of new diagnostic/prognostic and therapeutic target for lung cancer as well as for many other types of malignancies [8,9]. RNA binding motif 5 (RBM5, also called Luca15 or H37 [10,11]) maps to one end of this 19-gene deletion breakpoint, which is common to lung, breast and renal tumors [12].

At present, studies on the link of RBM5 and lung cancer are mainly confined to the cell lines. Most studies showed that the expression of RMB5 on cancer cells was virtually similar to that on the non-cancer cells. The possible reasons for this might be due to the long period of *in vitro* fostering and the changes of genetic expression [8,13,14]. Few studies on the expression and function of RBM5 on lung cancer tissues, especially on the tissues of primary lung adenocarcinoma, could be found [11,15,16]. Two groups examined the RBM5 expression on a small number of lung cancer tissues and found that most lung cancer, except a large cell carcinoma subtype that showed higher RBM5 expression, had remarkably lower expression of RBM5 [11,15]. In this study, we examined the RBM5 expression on 30 samples of lung adenocarcinoma patients in the hope to better understand the role and function of this cancer suppressor in the fostering and growing of lung adenocarcinoma.

RBM5 is an RNA-binding protein that has the ability to modulate apoptosis [17-19]. Overexpression of RBM5 sensitizes cells to certain apoptotic stimuli and induces apoptosis [17]. In addition, overexpression of RBM5, which is also involved in the regulation of alternative splicing, was shown to inhibit tumor growth and reduced the metastatic potential [9,20].

To further investigate the mechanism behind this modulation and sensitization process, we examined the expression of key apoptosis-associated genes in RBM5-overexpressing lung cancer cells. Our results showed that RBM5 significantly inhibited the growth of A549 cells both *in vitro* and *in vivo*, and induced apoptosis by increasing expression of cleaved caspase-3, caspase-9 and PARP proteins and reducing expression of Bcl-2 protein. Moreover, we propose a novel animal model set up in BALB/c nude mice treated with attenuated Salmonella as a vector carrying plasmids to determine RBM5 function *in vivo.*

## Methods

### Specimen collection

This study was approved by the Institutional Review Board of the Second Affiliated Hospital of Jilin University, Changchun, China. Thirty pairs of tissues and the information of disease history were collected from the patients, who had been diagnosed with primary lung adenocarcinoma. All participants underwent surgery and provided written informed consent. Samples were snap-frozen in liquid nitrogen at the time of study and stored at −80°C until RNA or protein was extracted following the routine protocol. All cases were reevaluated by pathologists for confirming tumor histology and tumor content.

### Cell culture

A549 cell line was purchased from the American Tissue Type Collection (Manassas, VA). Cells were grown in RPMI 1640 supplemented with 10% fetal bovine serum as previously described [21].

### Transfections

A549 cells were transiently transfected with pcDNA3.1 or pcDNA3.1-RBM5 plasmids using the Lipofectamine 2000 reagent (Roche, Switzerland) for the indicated times according to the manufacturer’s instruction. Briefly, 1 × 10^5^ cells were seeded into six-well plates containing an antibiotic-free medium and incubated overnight. For each well, 2 μg DNA (pcDNA3.1 or pcDNA3.1-RBM5) was mixed with 95 μl RPMI-1640 with 10% fetal bovine serum. The mixture was then combined with a solution of 5 μl Lipofectamine (Roche, Switzerland) in 95 μl RPMI-1640 with 10% fetal bovine serum. After a 20-min incubation period at room temperature, the mixture was applied to the cells in an appropriate volume of OPTI-MEM I so as to achieve a final volume of 2 ml. Then the cells were cultured for an additional 24 h/48 h at 37°C before analysis.

### Proliferation assay in A549 cells

Cell proliferation assay was evaluated using MTT according to the manufacturer’s instruction. Briefly, A549 cells transfected with pcDNA3.1 or pcDNA3.1-RBM5 plasmids were seeded in a 96-well plate at a density of 1 × 10^4^ cells per well (0 hour). At 24 h and 48 h, 20 μl of 5 mg/ml MTT in PBS was added, and the cells were subsequently incubated for 4 h at 37°C in 5% CO_2_. Cells were then washed twice with PBS, and the precipitate was solubilized in 150 μl of 100% dimethylsulfoxide (Sigma, USA) by shaking for 5 min. Absorbance was measured using a microplate reader (Bio-Rad, Richmond, CA) at a wavelength of 570 nm. All experiments were carried out in triplicate.

### Reverse transcription-polymerase chain reaction (RT- PCR)

Total RNAs were isolated using Trizol (Invitrogen, USA) according to the manufacturer’s instruction. Reverse transcription was performed with 3 μg of total RNA at a final volume of 10 μl, containing 10 mM dNTP, 0.5 μg oligo dT, 20 U RNasin and 200 U M-MLV reverse transcriptase (Promega Corp., USA). The primer sequences were RBM5: 5′-GCACGACTATAGGCATGACAT-3′ and 5′-AGTCAAACTTGTCTGCTCCA-3′, GAPDH: 5′-GAAGGTGAAGGTCGGAGTC3′ and 5′-GAAGATGGTGATGGGATTTC-3′. PCR was performed at 95°C for 3 min and 25–30 cycles of 95°C for 30 s, 55°C for 30 s, 72°C for 1 min and 72°C for 10 min. All densitometry scanning was carried out using ImageMaster VDS Software (Pharmacia Biotech, USA).

### Protein extraction and Western blot

Total cellular proteins from both lung tissues and A549 cells were extracted according to the previous study [22]. Protein samples (50 ug) were then separated by SDS-PAGE and transferred onto a PVDF membrane (Millipore, Bedford, MA). The primary antibodies were rabbit anti-human RBM5, Bcl-2, PARP, cleaved-PARP, caspase-3, cleaved caspase-3 and β-actin antibodies from Abcam (MA, USA). The primary antibodies of rabbit anti-human caspase-9 and cleaved caspase-9 antibodies were from Cell Signaling Technology (USA). The second antibody was a goat anti-rabbit IgG-HRP from Santa Cruz Biotechnology (CA, USA). Western blot was carried out as previously described [22]. The protein bands were visualized by SuperSignal West Pico Chemiluminescent Substrate (Pierce, Rockford, IL, USA), and the membranes were subjected to X-ray autoradiography. Band intensities were determined with Quantity One software (Bio-Rad, Hercules, CA, USA). Furthermore, we confirmed the reproducibility of the experiments at least three times.

### Flow cytometry analysis of apoptosis

A549 cells were transfected with pcDNA3.1 or pcDNA3.1-RBM5 plasmids using the Lipofectamine 2000 reagent (Roche, Switzerland) for 48 h, followed by harvesting, counting (1 × 10^6^ cells) and resuspending in 100 μl of phosphate-buffered saline (PBS). Afterward, 5 μl of AnnexinV (1 μg/ml) (Beckman Coulter, Fullerton, CA) was added and incubated at RT for 15 min, then 10 μl of propidium iodide (PI 1 μg/ml) was added and incubated for additional 5 min at room temperature in the dark. Finally, the cells were subjected to flow cytometry (FCM) to measure the apoptosis rate with an Epics-XL-MCL flow cytometer (Beckman Coulter, USA).

### Tumor growth analysis in A549 Xenograft

The models of A549 xenografts were established using BALB/c nude mice (6 weeks old) that were acquired from the Institute of Zoology, Chinese Academy of Sciences (Beijing, China). Use of animals was in accordance with Animal Care guidelines, and the protocol was approved by Jilin University Animal Care Committee. Cultured cells were washed and resuspended in phosphate-buffered saline (PBS). The suspension (5 × 10^6^ cells in 150 μl/ per mouse) was inoculated subcutaneously into the right flanks of nude mice. The sizes of the tumors were measured starting from day 7 after cell injection until day 21 using calipers. These tumor-bearing mice were then divided randomly into two groups (six mice per group): (1) PcDNA3.1 as the control group; (2) pcDNA3.1-RBM5 as the RBM5 group. Plasmids were carried by bioengineered attenuated strains of *Salmonella enterica serovar typhimurium* using electrotransfection as previously described [22,23]. Briefly, plasmids were electrotransfected into *Salmonella typhi* Ty21a competent cells before use. Mice in each of these groups were inoculated with bacteria with control vector (pcDNA3.1) or pcDNA3.1-RBM5 plasmids [10^8^ colony-forming units (CFU) per 50 μl] via tail vein injection two times (on day 28 and 35). Tumors were measured using calipers every 4 days for 42 days in total, and the data were plotted using the Kaplan-Meier method to analyze the tumor growth curves. In addition, the wet weight and sizes of tumors were measured when mice were killed. To ensure the tumor-preferable distribution of the bacteria, an additional pilot study was performed before the above-described animal experiments. Tissue samples of the primary tumor, liver, lung, spleen, heart and kidney from mice were used for bacterial distribution analysis on day 3 and 7 after injection of attenuated Salmonella carrying plasmid. Equal amounts of tissues were collected, minced, and homogenized. Afterward, the homogenized tissues were plated in triplicate onto Luria-Bertani agar containing ampicillin (100 mg/ml) for 24 h, followed by counting of bacterial colonies and CFU evaluation.

### Statistical analysis

The *χ*^2^ and Fisher’s exact tests were used to analyze the association of mutations with clinical characteristics. All *p* values were calculated based on a two-tailed hypothesis. *p* <0.05 was considered statistically significant.

## Results

### RBM5 expression was significantly decreased in primary lung adenocarcinoma

**Figure 1:**
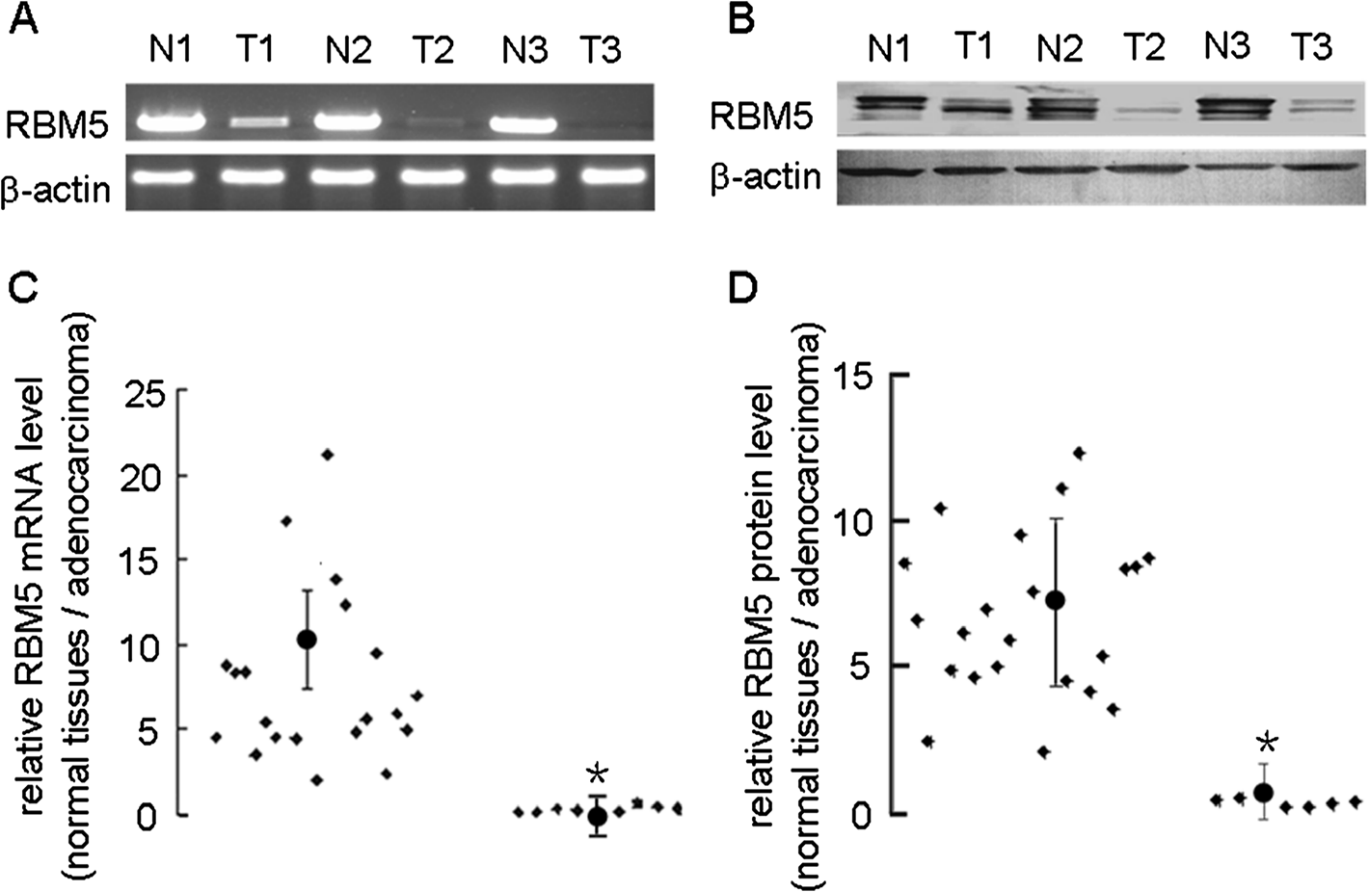
**Expression of RBM5 in human lung cancer tissues.(A)** Agarose gel of semi-quantitative RT-PCR data of RBM5 mRNA expression in representative samples from tumor and non-tumor specimens. Total RNA was isolated and subjected to semi-quantitative RT-PCR and quantified using Quantity One software. **(B)** Western blot of RBM5 protein expression in representative samples from tumor and non-tumor specimens. Total cellular protein was extracted, subjected to Western blot analysis and quantified using Quantity One software. **(C)** The ratio of RBM5 mRNA expression level in normal lung tissues (N) to that in lung adenocarcinoma tissues (T). *χ2 = 2.814, *p* < 0.05 indicates significant difference as compared to the groups whose ratio > 1. **(D)** The ratio of RBM5 protein expression level in non-tumor tissue (N) to that in lung adenocarcinoma tissues (T) from 30 patients with lung adenocarcinoma. *χ2 = 2.963, *p* < 0.05 indicates significant difference as compared to the groups whose ratio > 1.

To assess the expression of RBM5 mRNA and protein in human lung adenocarcinoma, we performed RT-PCR and Western blot analysis on 30 pairs of primary lung tumor versus adjacent non-tumor tissues. Our results showed that the expression of both RBM5 mRNA and protein was decreased in lung tumor compared to that in the non-tumor counterpart (Figure 1C, *χ*2 = 2.814, *P* < 0.05; Figure 1D, *χ*2 = 2.963, *P* < 0.05). When the bands were quantitatively compared, expression of RBM5 mRNA was found to be lower in tumor compared to the non-tumor counterpart in the majority of the paired samples (except in 9 of the 30 patients, Figure 1A, C). Similarly, when the bands were quantitatively compared, the expression of RBM5 protein was found to be lower in tumor compared to the non-tumor counterpart in the majority of the paired samples (except in 7 of the 30 patients, Figure 1B, D). Our result confirms the previous finding that RBM5 expression was decreased in human lung adenocarcinoma tissues compared to that in the corresponding non-tumor tissues.

### Overexpression of RBM5 inhibits A549 cells proliferation

**Figure 2:**
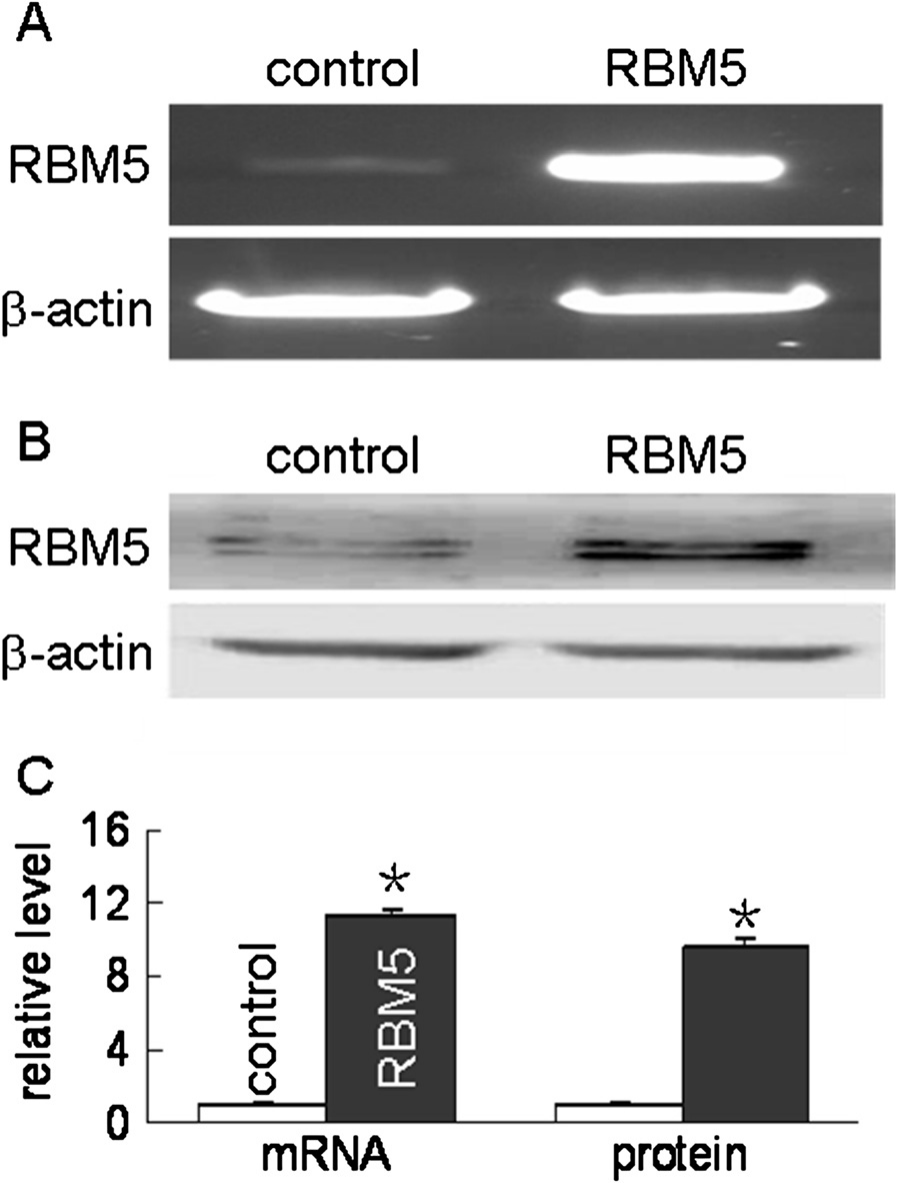
**Overexpression of RBM5 in A549 cells.** A549 cells were transfected with pcDNA3.1 or pcDNA3.1 RBM5 plasmids for 48 h. RT-PCR and Western blot were performed respectively to determine the RBM5 mRNA **(A)** and protein levels **(B)**. Data shown are means ± SD of three separate experiments **(C)**. *Significant difference as compared to the control (*p* < 0.01).

**Figure 3:**
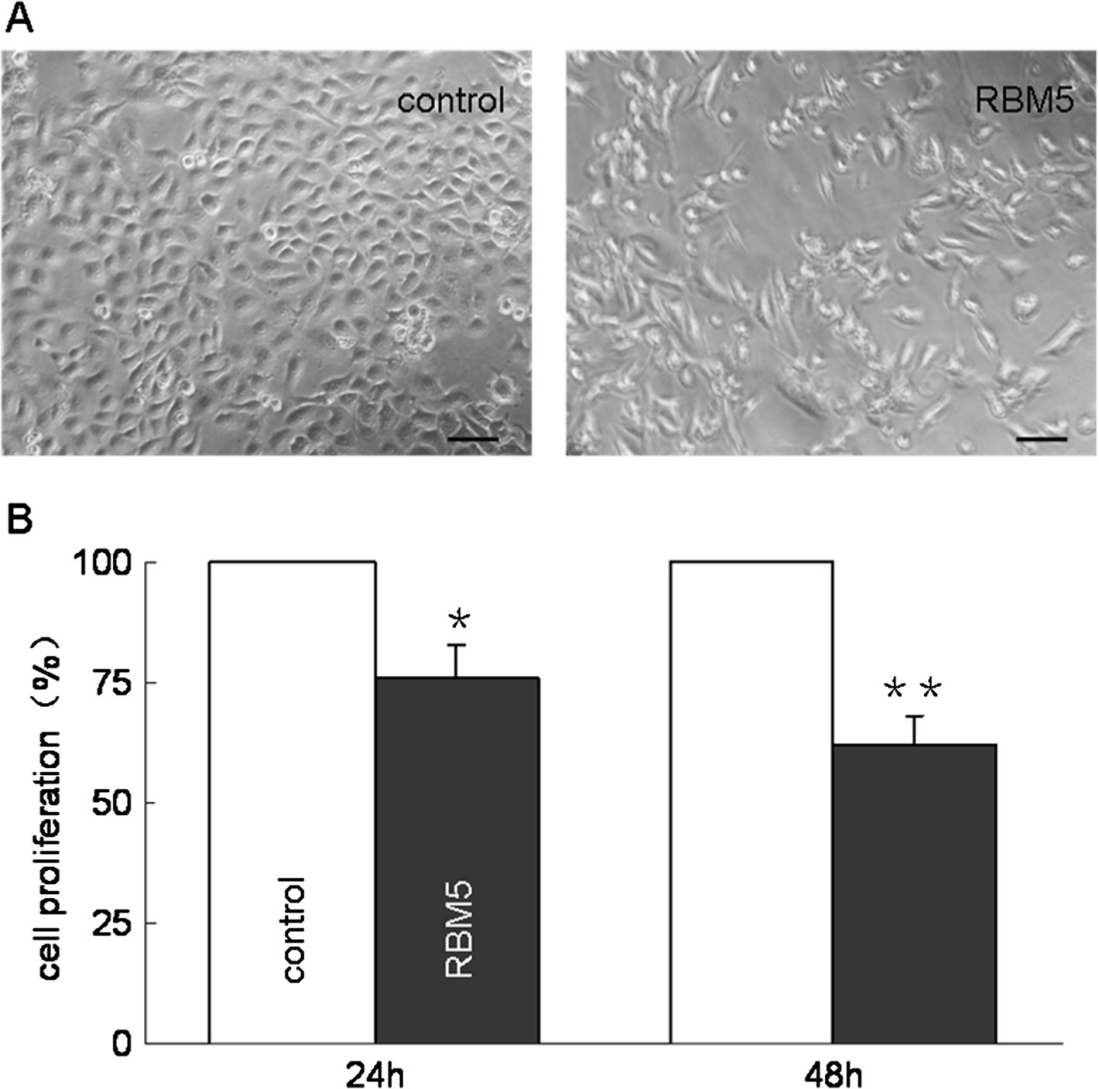
**Effect of RBM5 overexpression on A549 cells proliferation.** A549 cells were transfected with pcDNA3.1 or pcDNA3.1-RBM5 plasmids for up to 24 h and 48 h. Afterward, cell growth was observed under microscopy **(A)** and evaluated by MTT assay **(B)**. The data are presented as the ratio compared to the control (control = 100%). Data shown are means ± SD of three separate experiments. * *p* < 0.05, ** *p* < 0.01 indicate significant difference as compared to the control.

To further determine the relationship between RBM5 gene and tumor growth, we performed the proliferation assay in A549 cells to explore the effect of RBM5 overexpression on proliferation of tumor cells. We transiently transfected A549 cells with pcDNA3.1-RBM5, then the expression level of RBM5 in A549 cells was determined by RT-PCR and Western blot, presented in Figure 2. MTT assays were performed at 24 h and 48 h, respectively, after the transfection. Results showed that there was a significant inhibition of cell proliferation in RBM5-overexpressing A549 cells compared to that in controls (Figure 3 A, B). Along with the extension of the time, the inhibition rate was increased.

### Overexpression of RBM5 induces apoptosis in A549 cells

**Figure 4:**
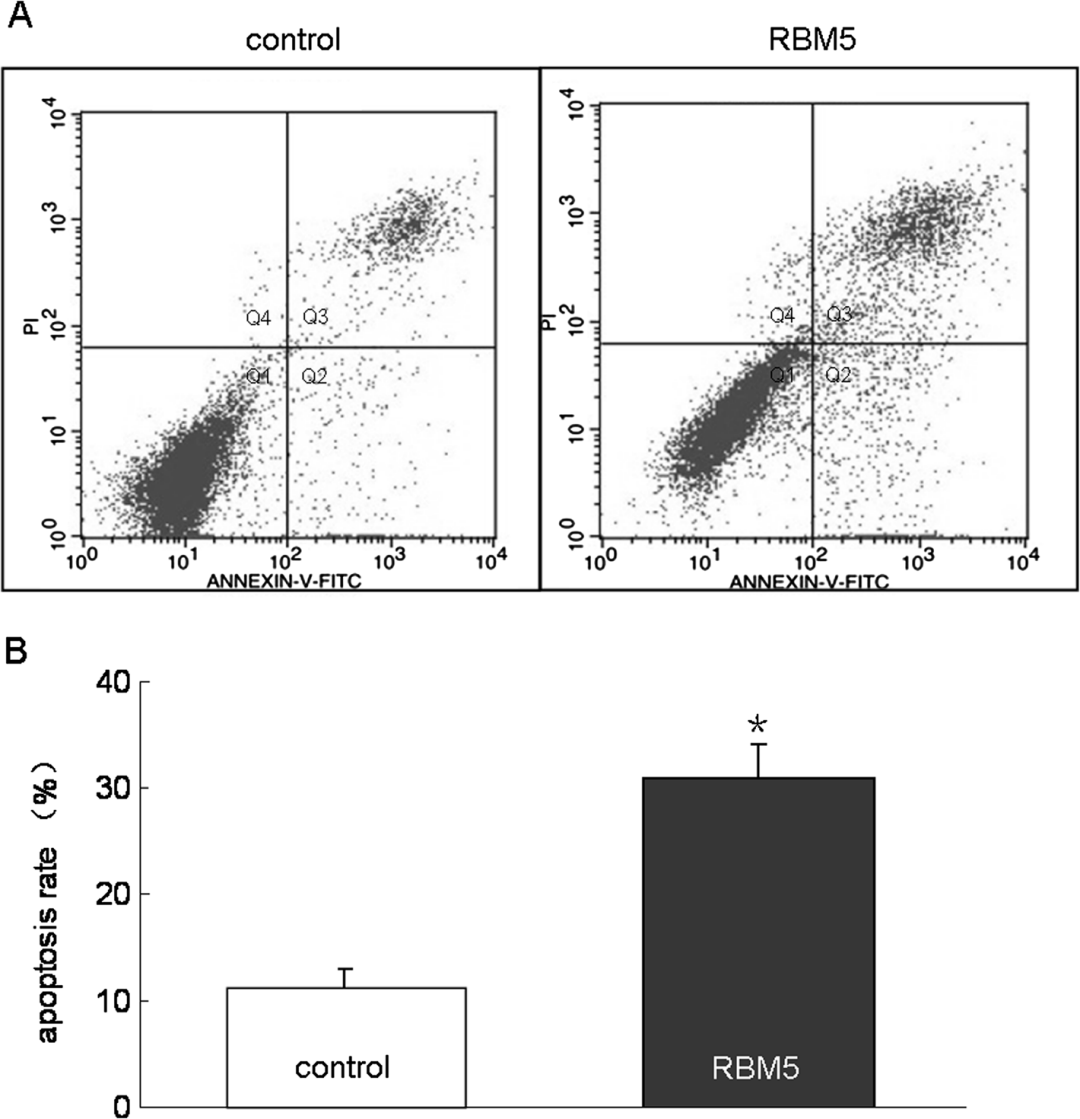
**Effect of RBM5 overexpression on apoptosis in A549 cells.** A549 cells were harvested at 48 h after transfection. PS flip to the outer nuclear membrane was monitored by staining cells with FITC-conjugated Annexin V, which binds to the PS and PI **(A)**. Data are presented as a flow cytomter dot plot. Q1, live cells; Q2, early apoptotic cells; Q3, late apoptotic cells; Q4, necrosis cells. **(B)** The representative data of FCM with Annexin V and PI staining for detecting apoptotic cells are shown in control and RBM5 transfected groups. *Significant difference as compared to the control (*p* < 0.05).

We next explored the mechanisms underlying growth inhibition by RBM5. To determine the contribution of cell death induced by RBM5, we employed FCM to detect apoptotic cells, which are characterized by phosphatidyl serine (PS) translocation on the outer cell membrane. Cells were double-stained with AnnexinV and PI after 48 h transfection. Representative raw FCM data are as follows: Cells transfected with RBM5 showed a higher proportion of early and late apoptosis (13.41% and 18.28%) as compared to the control cells (4.01% and 7.94%), respectively (Figure 4). The early and the late apoptotic cells were distributed in the Q2 and Q3 regions, respectively. The necrotic cells were located in the Q4 region. The result suggested that overexpression of RBM5 significantly increased apoptosis in lung cancer cells.

### RBM5 overexpression is associated with alteration of apoptosis-related genes

**Figure 5:**
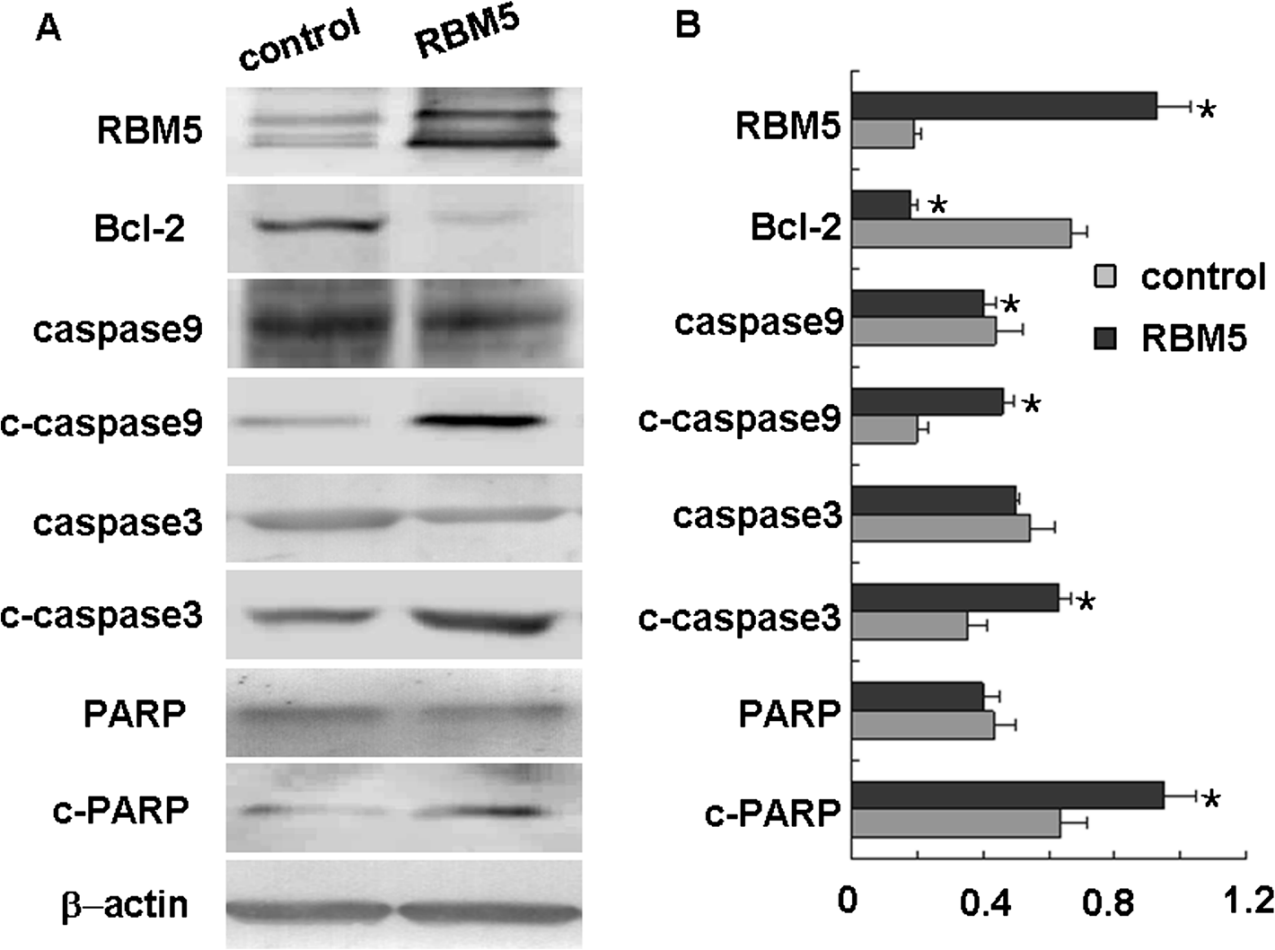
**Effect of RBM5 on the expression of apoptosis-related genes in A549 cells.** Cellular proteins were extracted from A549 cells transfected with pcDNA3.1 or pcDNA3.1-RBM5 plasmids for 48 h. **(A)** Expression of RBM5, Bcl-2, caspase-3, cleaved caspase-3, caspase-9, cleaved caspase-9, PARP and cleaved PARP were determined by Western blot. **(B)** Quantitative data from A. *Significant difference as compared to the control (*p* < 0.05).

To further study the possible molecular mechanisms underlying RBM5-induced cell apoptosis, we next examined the expression of some apoptosis-related genes including Bcl-2, caspase-3, caspase-9 and PARP. We observed that the expression of Bcl-2 protein was decreased significantly when RBM5 was overexpressed, while the expression of cleaved caspase-3, caspase-9 and PARP proteins was significantly increased in the RBM5 overexpressing cells as compared to that in the control cells. Accordingly, the expression of caspase-9 protein was slightly decreased in RBM5 overexpressing cells as compared to that in the control cells, while the expression of caspase-3 and PARP had no significant difference in either group (Figure 5). This result again suggested that RBM5, by decreasing Bcl-2 expression, could induce caspase-3, caspase-9, PAPP cleavage and promoted apoptosis.

### Assessment on transfection of the attenuated Salmonella typhi Ty21a carrying plasmids into A549 xenograft BALB/c nude mice

**Figure 6:**
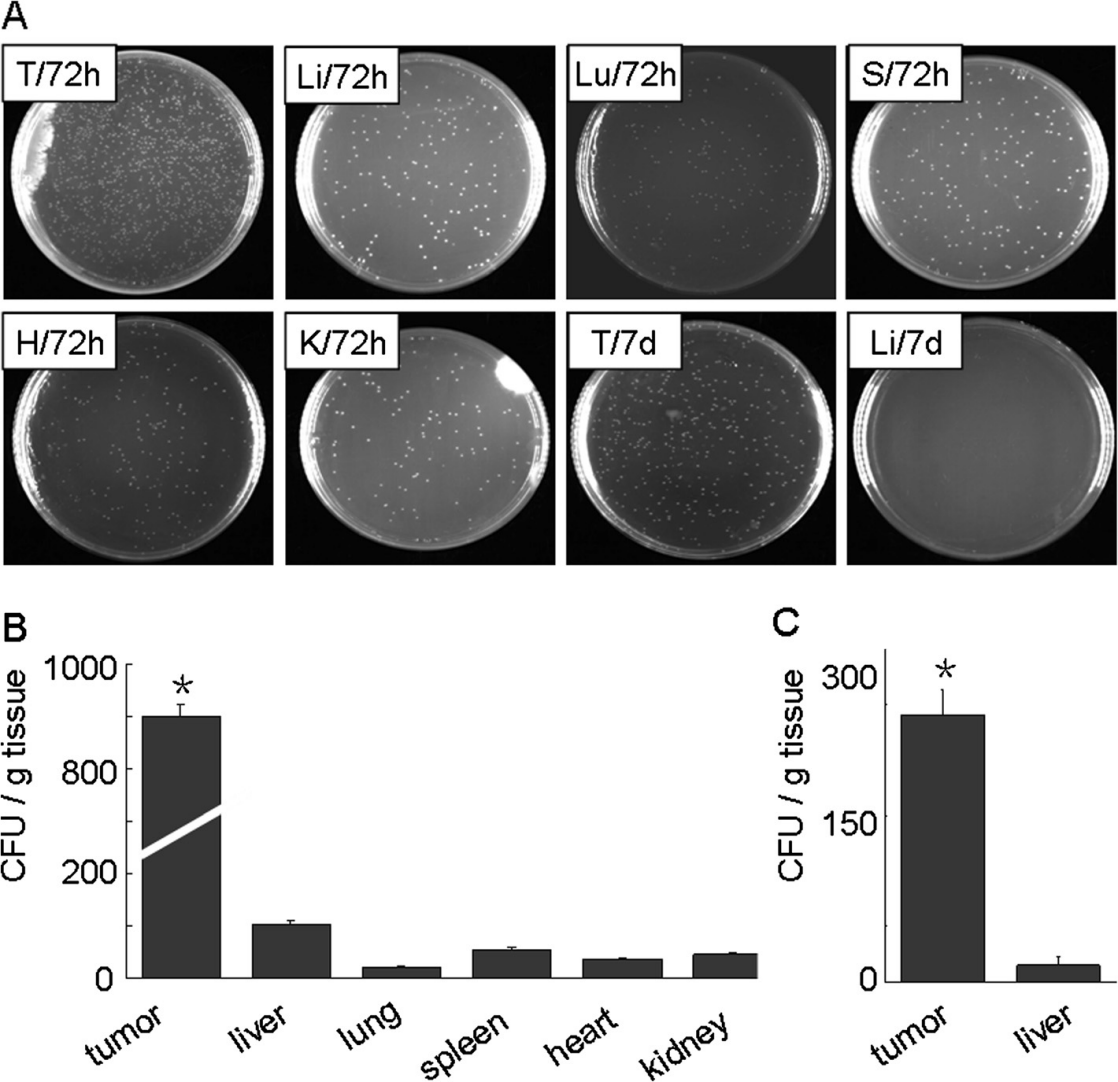
**Assessment of tumor-preferable distribution of the bacteria.** Tissue samples from the lung tumor xenografts, liver, lung, spleen, heart and kidney of three tumor-bearing mice treated with bacteria carrying vector control were collected for bacterial distribution analysis. Tissue (100 mg) from either tumors or each organ in 3 ml of PBS was homogenized well, and the homogenized tissue was plated onto Luria-Bertani agar. The result was observed after 14 h. **(A)** Representative images of the plates planted with different tissues at day 3 (72 h) and day 7 (148 h) after bacterial injection. T, tumor xenograft; Li, liver; Lu, lung; S, spleen; H, heart; K, kidney. **(B)** and **(C)** Quantitative analysis of the bacterial counts by CFU per gram of tissue at day 3 **(B)** and day 7 **(C)** after bacterial injection. *Significant difference in tumor xenograft group as compared to other group (*p* < 0.05).

To further test our hypothesis, the model of A549 xenograft was established as described in Materials and methods. At the 28th day after implantation, the tumor-bearing mice were inoculated with bacteria carrying the pcDNA3.1 vector tumor with 10^8^ CFU in 50 μl PBS per mouse. To ensure that attenuated *Salmonella typhi* Ty21a carrying plasmids preferentially localized in the tissue, we monitored the kinetics of bacterium distribution in the xenograft tumor and different organs of the tumor-bearing area at certain post-injection times (Figure 6A). On day 3 (72 h) after injection, bacteria could be found predominantly in the tumors compared to other organs (Figure 6 A, B; *p* < 0.05), which indicated the tumor-targeting character of attenuated *Salmonella typhi* Ty21a. We found that the concentration of bacteria gradually decreased in the tumors, however, which was found decrease or vanish significantly in other organs on day 7 after injection (Figure 6 A, C). We concluded that the bacteria could be accumulated in the tumors in the course of treatment since we injected the mice every 7 days from day 28 to day 42. Quantitative analysis of the bacteria by CFUs as described in Materials and methods (Figure 6B and C) confirmed the predominant distribution of bacteria in the tumor tissues.

### Suppression of tumor growth by RBM5 in A549 xenograft tumors and the alteration of apoptosis-related genes

**Figure 7:**
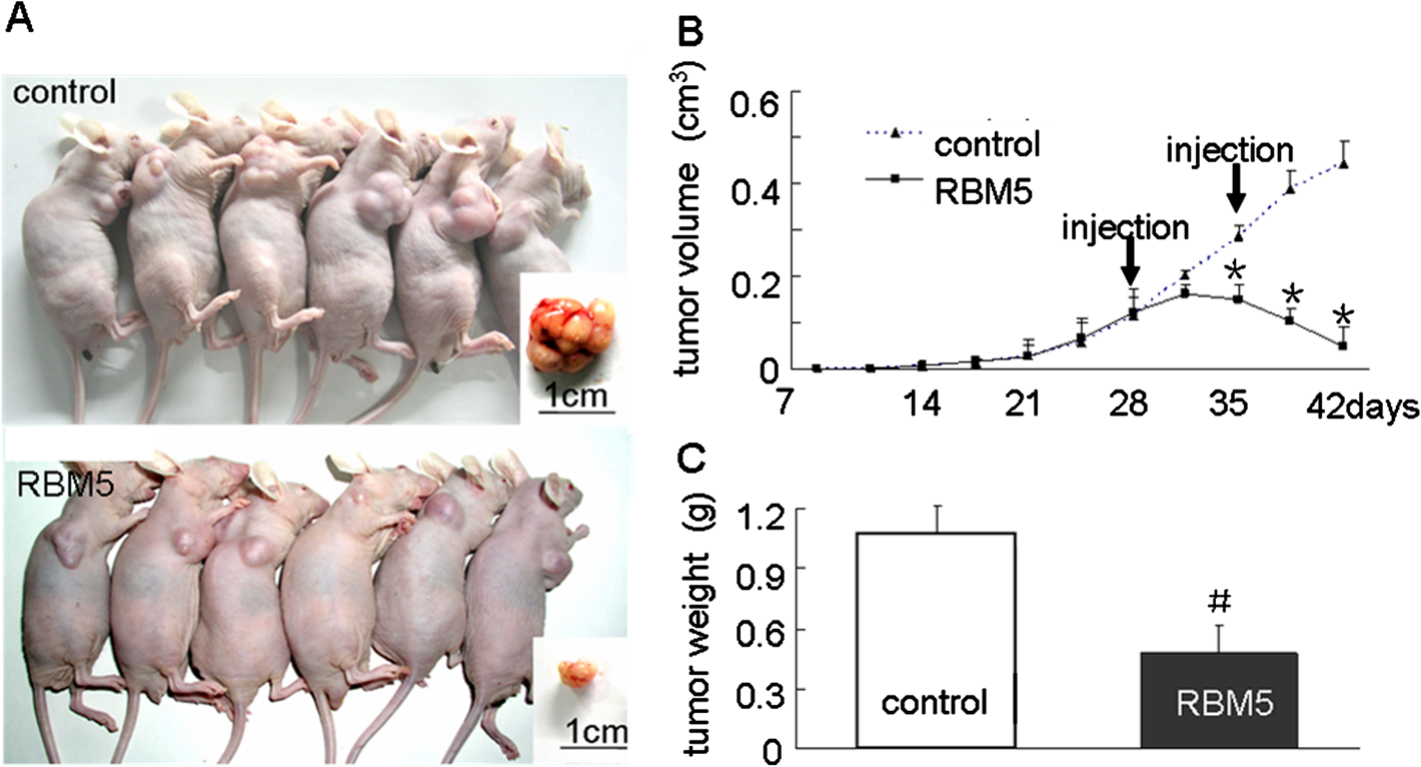
**RBM5 inhibits tumor growth *in vivo*.** Tumor-bearing mice were treated with attenuated Salmonella carrying pcDNA3.1 or pcDNA3.1-RBM5 by injection two times (on day 28 and 35). **(A)** Comparison of tumor size in two groups on day 42 after implantation. **(B)** Tumor growth curve. Tumor sizes were measured two times every week from day 7 to 42 after implantation. **(C)** Tumor wet weights were measured when the mice were killed on day 42 after implantation. *Significant difference as compared to the control (*p* < 0.05); #Significant difference as compared to the control (*p* < 0.01).

The potential therapeutic effect on lung adenocarcinoma xenograft growth by RBM5 was examined in tumor-bearing mice treated with bacteria carrying RBM5 plasmid (Figure 7A). Dynamic tumor growth was monitored from day 7 to day 42 after injection. We showed that, while the sizes of the tumor xenografts between RBM5 and control groups were similar before day 28, the growth of tumor xenografts in the mice treated with RBM5 decreased after the 28^th^ day (Figure 7B). In addition, the weight of the tumor xenografts from mice treated with RBM5 became significantly lighter than that in the control group at the 42^nd^ day when the mice were killed (Figure 7C). This result suggested that accumulative and stable expression of RBM5 in A549 xenograft BALB/c nude mice significantly retarded the tumor growth rate *in vivo*. Moreover, we set up a novel animal model using BALB/c nude mice treated with attenuated Salmonella as a vector carrying plasmids to determine RBM5 function in vivo.

**Figure 8:**
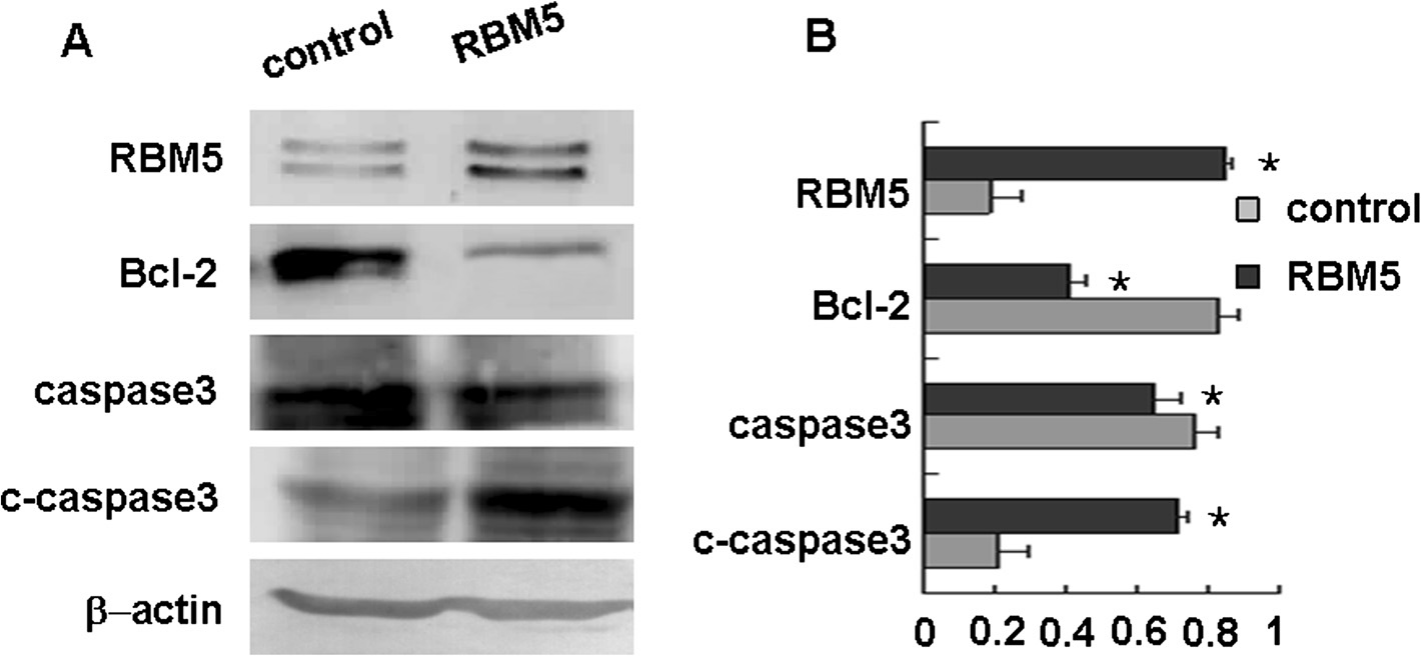
**Effect of RBM5 on the expression of apoptosis-related genes in A549 xenograft BALB/c nude mice.** Proteins were extracted from tumor tissues treated with attenuated salmonella carrying pcDNA3.1 or attenuated salmonella carrying pcDNA3.1-RBM5. **(A)** Expression of RBM5, Bcl-2, caspase-3 and cleaved caspase-3 were determined by Western blot. **(B)** Quantitative data from A. *Significant difference as compared to the control (*p* < 0.05).

To further explore whether it possesses the same molecular mechanisms underlying RBM5 functions on both in vitro and in vivo, we examined the expression of Bcl-2 and caspase-3 proteins in A549 xenograft. Similarly, we found that the expression of Bcl-2 was significantly decreased in A549 xenograft BALB/c nude mice treated with RBM5 as compared to the control mice, while the expression of cleaved caspase-3 was significantly increased in RBM5-treated mice (Figure 8). This result, consistent with the cell line study, suggested again that RBM5 inhibited Bcl-2 expression, triggered cleavage of caspase-3, promoted apoptosis and suppressed the tumor growth *in vivo.*

## Discussion

There is increasing evidence suggesting that RBM5 plays an important role in lung cancer occurrence and development; nevertheless, there are few studies reporting the RBM5 expression in lung cancer tissues and tumor cell lines. In a very small cohort of eleven specimens, it was observed that the six tumor samples with the most significantly reduced RBM5 mRNA levels were of the squamous type [11,15,16] whereas three of the nine with less significantly reduced RBM5 mRNA levels were adenocarcinomas. The one tumor sample with no change in RBM5 mRNA expression compared to its non-tumor tissue was an adenocarcinoma, while the one tumor sample that had more RBM5 mRNA than its non-tumor tissue was a large cell carcinoma [11,15]. These results suggest that RBM5 gene expression is related to histologic subtype in NSCLC. Moreover, RBM5 mRNA was downregulated in spontaneously developing human tumors such as human schwannomas [24] and in transformed cells, such as ras-transformed Rat-1 rat embryonic fibroblastic cells [25]. Consistent with the above findings, our study confirms previous study in terms of the RBM5 expression in lung adenocarcinoma and further suggests that RBM5 plays a critical role in the occurrence and development in lung carcinoma.

RBM5 is a putative tumor suppressor. The gene encodes a number of alternative RNA splice variants with differing abilities to enhance, sensitize or suppress apoptosis [13,17,26-28]. Thus, it is likely that the ability to modulate apoptosis is central to the putative tumor suppressor activity of RBM5. Despite increasing evidence showing the activity of the apoptotic modulator of RBM5 in lung cancer development, the detailed mechanism is still largely unknown. In order to further confirm the fact and investigate the potential molecular mechanism underlying the tumor suppressive activity of RBM5, we observed the effects of RBM5 on A549 cell growth and apoptosis with RBM5 overexpression. Our results showed that RBM5 overexpression significantly inhibited cell proliferation and induced early and late apoptosis in A549 cells, which was associated with downregulation of Bcl-2 and upregulation of cleaved caspase-3, -9 and PARP. Similar studies were reported in other groups [8,17,28]. Oh et al. showed that the cyclin A and phosphorylated RB levels were decreased, whereas expression of Bax protein was increased in RBM5-transfected A549 cells; this led to changes in the mitochondrial membrane potential, cytochrome c release into the cytosol, and enhanced caspase-9 and caspase-3 activities, thereby inducing apoptosis [8]. Note that Bcl-2 expression had no change in Oh’s study; a possible reason for the difference between our and this study might be the different transfection system. Tobayashi et al. demonstrated that overexpression of RBM5 enhanced p53-mediated inhibition of cell growth and colony formation in different cell lines. Expression of RBM5 augmented p53 transcriptional activity in reporter gene assays and resulted in increased mRNA and protein levels for endogenous p53 target genes [19]. In all, our study confirms previous findings and demonstrates the activity of the apoptotic modulator associated with RBM5.

In order to confirm our *in vitro* results, the lung adenocarcinoma transplantation *in vivo* model was made by injecting A549 cells onto the back of immunocompromised mice. Our results indicated that overexpression of RBM5 reduced the volume and weight of transplanted lung tumors significantly. Through bacterial distribution analysis, the characteristics of replicating preferentially in tumor cells for the *Salmonella typhi* Ty21a were identified. Previous study demonstrated that Salmonella could be infected and preferentially accumulated in the tumor xenograft *in vivo*, with a tumor/normal tissue-retaining ratio of approximately 1,000:1 [29]. Attenuated *Salmonella typhi* Ty21a is a facultative anaerobic bacterium that was capable of replicating preferentially in tumor cells and was used for developing proteins therapeutic to several different tumors [30-33]. We showed that a large amount of the *Salmonella typhi* Ty21a still remained in the tumor tissues at the later stage of treatment. This suggested that expression of RBM5 protein was maintained and continued to be effective in suppressing tumor growth throughout the whole process. The attenuated *Salmonella typhi* Ty2la bacteria approaches in our study demonstrated an important role of RBM5 in inhibition of tumor growth. Moreover, our *in vivo* results further confirmed the molecular mechanism underlying RBM5-induced apoptosis might be by means of inhibiting expression of bcl-2 and triggering the cleavage of caspases. In our *in vivo* assay, the transplant tumor model was established using A549 cells in both the experimental and control groups. When compared with Oh et al.’s study [8], the benefit of accumulative and relatively stably maintained expression of RBM5 in tumors in our *in vivo* study could better reflect the potential therapeutic role of RBM5 in effective inhibition of lung cancer cell growth. Thus, our *in vivo* study, which demonstrated the anti-tumor effect of RBM5 significantly, may be more convincing and promising, which demonstrated the anti-tumor effect of RBM5 significantly.

## Conclusion

In summary, our results showed that RBM5 significantly inhibits tumor growth and induces apoptosis of lung cancer cells by reducing the expression of Bcl-2, subsequently inducing the expression of cleaved caspase-3, cleaved caspase-9 and cleaved PARP. More importantly, accumulative and stable expression of RBM5 in tumors significantly inhibits the tumor growth rate *in vivo.* Finally, RBM5 might be used as a potential biomarker or target for lung cancer diagnosis and chemotherapy.

## Abbreviations

NSCLC, non-small cell lung cancer; RBM5, RNA binding motif 5; PS, phosphatidyl serine.

## Competing interests

The authors declare that they have no competing interests.

## Authors’ contributions

CS and LZ performed all the experiments and drafted the manuscript. WX collected and provided the tissues. JZ and KW contributed to the research design, the data collection and interpretation. KW oversaw the design of the study and was involved in the critical revision of the manuscript. BY oversaw the manuscript and gave a thorough revision. All authors have read and approved the final version of the manuscript.

## References

[B1] Siegel R Naishadham D Jemal A Cancer statistics, 2012 CA Cancer J Clin 2012 62 10 29 10.3322/caac.20138 22237781

[B2] Molina JR Yang P Cassivi SD Schild SE Adjei AA Non-small cell lung cancer: epidemiology, risk factors, treatment, and survivorship Mayo Clin Proc 2008 83 584 594 18452692 10.4065/83.5.584 PMC2718421

[B3] Wistuba II Mao L Gazdar AF Smoking molecular damage in bronchial epithelium Oncogene 2002 21 7298 7306 10.1038/sj.onc.1205806 12379874

[B4] Razmkhah M Doroudchi M Ghayumi SMA Erfani N Ghaderi A Stromal cell-derived factor-1 (SDF-1) gene and susceptibility of Iranian patients with lung cancer Lung Cancer 2005 49 311 315 10.1016/j.lungcan.2005.04.014 15955592

[B5] Alberg AJ Ford JG Samet JM Epidemiology of lung cancer - ACCP evidence-based clinical practice guidelines (2nd edition) Chest 2007 132 29s 55s 10.1378/chest.07-1347 17873159

[B6] Angeloni D Molecular analysis of deletions in human chromosome 3p21 and the role of resident cancer genes in disease Brief Funct Genomic Proteomic 2007 6 19 39 10.1093/bfgp/elm007 17525073

[B7] Ying J Poon FF Yu J Geng H Wong AH Qiu GH Goh HK Rha SY Tian L Chan AT DLEC1 is a functional 3p22.3 tumour suppressor silenced by promoter CpG methylation in colon and gastric cancers Br J Cancer 2009 100 663 669 10.1038/sj.bjc.6604888 19156137 PMC2653732

[B8] Oh JJ Razfar A Delgado I Reed RA Malkina A Boctor B Slamon DJ 3p21.3 tumor suppressor gene H37/Luca15/RBM5 inhibits growth of human lung cancer cells through cell cycle arrest and apoptosis Cancer Res 2006 66 3419 3427 10.1158/0008-5472.CAN-05-1667 16585163

[B9] Oh JJ Taschereau EO Koegel AK Ginther CL Rotow JK Isfahani KZ Slamon DJ RBM5/H37 tumor suppressor, located at the lung cancer hot spot 3p21.3, alters expression of genes involved in metastasis Lung Cancer 2010 70 253 262 10.1016/j.lungcan.2010.02.012 20338664

[B10] Timmer T Terpstra P van den Berg A Veldhuis PMJF Ter Elst A Voutsinas G Hulsbeek MMF Draaijers TG Looman MWG Kok K A comparison of genomic structures and expression patterns of two closely related flanking genes in a critical lung cancer region at 3p21.3 Eur J Hum Genet 1999 7 478 486 10.1038/sj.ejhg.5200334 10352938

[B11] Oh JJ West AR Fishbein MC Slamon DJ A candidate tumor suppressor gene, H37, from the human lung cancer tumor suppressor locus 3p21.3 Cancer Res 2002 62 3207 3213 12036935

[B12] Senchenko VN Liu J Loginov W Bazov I Angeloni D Seryogin Y Ermilova V Kazubskaya T Garkavtseva R Zabarovska VI Discovery of frequent homozygous deletions in chromosome 3p21.3 LUCA and AP20 regions in renal, lung and breast carcinomas Oncogene 2004 23 5719 5728 10.1038/sj.onc.1207760 15208675

[B13] Sutherland LC Edwards SE Cable HC Poirier GG Miller BA Cooper CS Williams GT LUCA-15-encoded sequence variants regulate CD95-mediated apoptosis Oncogene 2000 19 3774 3781 10.1038/sj.onc.1203720 10949932

[B14] ter Elst A Hiemstra BE van der Vlies P Kamminga W van der Veen AY Davelaar I Terpstra P Meerman GJT Gerbens F Kok K Buys CHCM Functional analysis of lung tumor suppressor activity at 3p21.3 Genes Chromosomes Cancer 2006 45 1077 1093 10.1002/gcc.20367 16958100

[B15] Angeloni D ter Elst A Wei MH van der Veen AY Braga EA Klimov EA Timmer T Korobeinikova L Lerman MI Buys CH Analysis of a new homozygous deletion in the tumor suppressor region at 3p12.3 reveals two novel intronic noncoding RNA genes Genes Chromosomes Cancer 2006 45 676 691 10.1002/gcc.20332 16607615

[B16] Ramaswamy S Ross KN Lander ES Golub TR A molecular signature of metastasis in primary solid tumors Nat Genet 2003 33 49 54 10.1038/ng1060 12469122

[B17] Rintala-Maki ND Sutherland LC LUCA-15/RBM5, a putative tumour suppressor, enhances multiple receptor-initiated death signals Apoptosis 2004 9 475 484 15192330 10.1023/B:APPT.0000031455.79352.57

[B18] Sutherland LC Lerman M Williams GT Miller BA LUCA-15 suppresses CD95-mediated apoptosis in Jurkat T cells Oncogene 2001 20 2713 2719 10.1038/sj.onc.1204371 11420683

[B19] Kobayashi T Ishida J Musashi M Ota S Yoshida T Shimizu Y Chuma M Kawakami H Asaka M Tanaka J p53 transactivation is involved in the antiproliferative activity of the putative tumor suppressor RBM5 Int J Cancer 2011 128 304 318 10.1002/ijc.25345 20309933

[B20] Akhtar MJ Ahamed M Khan MA Alrokayan SA Ahmad I Kumar S Cytotoxicity and apoptosis induction by nanoscale talc particles from two different geographical regions in human lung epithelial cells Environ Toxicol 2012 10.1002/tox.21766 22331707

[B21] Mu YM Oba K Yanase T Ito T Ashida K Goto K Morinaga H Ikuyama S Takayanagi R Nawata H Human pituitary tumor transforming gene (hPTTG) inhibits human lung cancer A549 cell growth through activation of p21(WAF1)/CIP1 Endocr J 2003 50 771 781 10.1507/endocrj.50.771 14709851

[B22] Lin GM Zhao LJ Yin F Lan RF Li LB Zhang XM Zhang H Yang BX TCF3 inhibits F9 embryonal carcinoma growth by the down-regulation of Oct4 Oncol Rep 2011 26 893 899 21725619 10.3892/or.2011.1376

[B23] Lerman MI Minna JD The 630-kb lung cancer homozygous deletion region on human chromosome 3p21.3: identification and evaluation of the resident candidate tumor suppressor genes. The International Lung Cancer Chromosome 3p21.3 Tumor Suppressor Gene Consortium Cancer Res 2000 60 6116 6133 11085536

[B24] Welling DB Lasak JM Akhmametyeva E Ghaheri B Chang LS cDNA microarray analysis of vestibular schwannomas Otol Neurotol 2002 23 736 748 10.1097/00129492-200209000-00022 12218628

[B25] Edamatsu H Kaziro Y Itoh H LUCA15, a putative tumour suppressor gene encoding an RNA-binding nuclear protein, is down-regulated in ras-transformed Rat-1 cells Genes Cells 2000 5 849 858 10.1046/j.1365-2443.2000.00370.x 11029660

[B26] Kotlajich MV Hertel KJ Death by splicing: tumor suppressor RBM5 freezes splice-site pairing Mol Cell 2008 32 162 164 10.1016/j.molcel.2008.10.008 18951082

[B27] Farina B Fattorusso R Pellecchia M Targeting Zinc Finger Domains with Small Molecules: Solution Structure and Binding Studies of the RanBP2-Type Zinc Finger of RBM5 ChemBioChem 2011 12 2837 2845 10.1002/cbic.201100582 22162216 PMC3408030

[B28] Maarabouni MM Williams GT The antiapoptotic RBM5/LUCA-15/H37 gene and its role in apoptosis and human cancer: research update ScientificWorldJournal 2006 6 1705 1712 17195868 10.1100/tsw.2006.268 PMC1825760

[B29] Pawelek JM Low KB Bermudes D Tumor-targeted Salmonella as a novel anticancer vector Cancer Res 1997 57 4537 4544 9377566

[B30] Cryz SJ Jr Vanprapar N Thisyakorn U Olanratmanee T Losonsky G Levine MM Chearskul S Safety and immunogenicity of Salmonella typhi Ty21a vaccine in young Thai children Infect Immun 1993 61 1149 1151 8432597 10.1128/iai.61.3.1149-1151.1993 PMC302854

[B31] Kimura H Zhang L Zhao M Hayashi K Tsuchiya H Tomita K Bouvet M Wessels J Hoffman RM Targeted therapy of spinal cord glioma with a genetically modified Salmonella typhimurium Cell Prolif 2010 43 41 48 10.1111/j.1365-2184.2009.00652.x 19922490 PMC4299869

[B32] Ji K Wang B Shao YT Zhang L Liu YN Shao C Li XJ Li X Hu JD Zhao XJ Synergistic suppression of prostatic cancer cells by coexpression of both murine double minute 2 small interfering RNA and wild-type p53 gene In Vitro and In Vivo J Pharmacol Exp Ther 2011 338 173 183 10.1124/jpet.111.180364 21444629

[B33] Shao Y Liu Y Shao C Hu J Li X Li F Zhang L Zhao D Sun L Zhao X Enhanced tumor suppression in vitro and in vivo by co-expression of survivin-specific siRNA and wild-type p53 protein Cancer Gene Ther 2010 17 844 854 10.1038/cgt.2010.41 20706288 PMC3915357

